# Laparoscopic Completion Cholecystectomy: An Audit from the Americas Hepato-Pancreato-Biliary Association (AHPBA) Caribbean Chapter

**DOI:** 10.7759/cureus.11126

**Published:** 2020-10-24

**Authors:** Shamir O Cawich, Sanjib K Mohanty, Kimon Bonadie, Lindberg Simpson, Rene Ramnarace, Patrick Fa Si Oen, Yardesh Singh, Vijay Naraynsingh, Wesley Francis

**Affiliations:** 1 Surgery, University of the West Indies, St. Augustine, TTO; 2 Surgery, Cayman Islands Hospital, Georgetown, CYM; 3 Surgery, Cayman Islands National Hospital, Grand Cayman, CYM; 4 Surgery, Kingston Public Hospital, Kingston, JAM; 5 Gastroenterology, Southern Medical Hospital, San Fernando, TTO; 6 Surgery, Princess Margaret Hospital, Curacao, CUW; 7 Surgery, Medical Associates Hospital, St. Joseph, TTO; 8 Surgery, Princess Margaret Hospital, Nassau, BHS

**Keywords:** cholecystectomy, laparoscopic, gallbladder, stump, remnant, completion, trinidad, caribbean

## Abstract

Objective

Removal of a gallbladder remnant occasionally becomes necessary when retained stones become symptomatic. Although the laparoscopic approach has been described, it is not yet considered the standard of care. We sought to determine the outcomes after completion cholecystectomies in the resource-poor setting within the Caribbean.

Methods

We carried out an audit of the databases from all hepatobiliary surgeons in the Anglophone Caribbean. We identified all patients who had completion cholecystectomy over the five-year period from July 1, 2012 to June 30, 2018. Retrospective chart review was performed to extract the following data: patient demographics, diagnoses, presenting complaints, operative details, morbidity, mortality, and clinical outcomes. Descriptive statistics were generated using Statistical Packaging for Social Sciences (SPSS), version 12.0 (SPSS Inc., Chicago IL)

Results

There were 12 patients who were subjected to laparoscopic completion cholecystectomy for acute cholecystitis (7), severe biliary pancreatitis (3), and chronic cholecystitis (2) secondary to stones in a gallbladder remnant. There were 10 women and two men at a mean age of 47.4 years (range 32-60; standard deviation (SD) +/-7.81; median 48; mode 52) and a mean body mass index (BMI) of 30.8 Kg/M^2^ (SD +/-3.81; range 26-38; median 29.5). The mean interval between the index operation and the completion operation was 14.8 months (SD +/- 12.3; range 1-48; median 13; mode 18). Five (42%) patients had their original cholecystectomy using the open approach. Five (42%) index operations were done on an emergent basis and the gallbladder remnant was deliberately left behind in three (25%) index operations. The completion cholecystectomies were all completed laparoscopically in 130.5 minutes (SD +/- 30.5; range 90-180, median 125; mode 125) without any conversions or mortality. There were two minor bile leaks that resolved without intervention through an indwelling drain.

Discussion

Completions cholecystectomy can be completed via the laparoscopic approach with good outcomes and acceptable morbidity and mortality rates. The patients derive the same advantages as elective cholecystectomies. Therefore, the laparoscopic approach, when performed by hepatobiliary surgeons with advanced laparoscopic expertise in specialized centers, should be the new standard of care.

## Introduction

Laparoscopic cholecystectomy is a basic operation that is performed routinely by general surgeons in the Caribbean. Many surgeons who encounter difficulty when dissecting Calot’s triangle will opt for a partial cholecystectomy to prevent iatrogenic bile duct injury (BDI) [1-3]. However, the gallbladder remnant may become diseased and then a completion operation to remove the remnant becomes necessary [4-7]. 

Although there are isolated reports of laparoscopic completion operations in the Caribbean [4,5], the true incidence of symptomatic remnants remains unknown. We sought to document the incidence of symptomatic gallbladder remnants in the Anglophone Caribbean.

These completion operations are technically difficult due to the presence of significant scarring and anatomic distortion after the original operation. Therefore, it is reasonable to expect that complications and conversion rates should be greater than with elective operations. A secondary aim of the study was to document the clinical outcomes when patients underwent completion operations in this resource-poor environment.

## Materials and methods

The Anglophone Caribbean is comprised of 17 countries, with a cumulative population of 6,426,914 persons [8]: Anguilla, Antigua & Barbuda, Bahamas, Barbados, Belize, British Virgin Islands, Cayman Islands, Dominica, Grenada, Guyana, Jamaica, Montserrat, St. Kitts & Nevis, St. Lucia, St. Vincent & the Grenadines, Trinidad & Tobago and Turks & Caicos. The University of the West Indies (UWI) was founded in 1948 to serve as a regional medical institution supported by and serving these 17 Caribbean countries [8]. Therefore, ethical approval for this study was sought from and granted by the UWI institutional review board. 

We also secured permission from three regional surgical societies to access a list of, and interview surgeons from, their general membership: the Caribbean College of Surgeons (CCOS) was founded in 2002 to promote surgical education for general surgeons practicing in the Caribbean; the Caribbean Association of Endoscopic Surgeons (CaSES) was founded in 2013 to support laparoscopic surgeons in the region, and the Caribbean Chapter of the Americas Hepato-Pancreato-Biliary Association (AHPBA) was founded in 2016 to support regional sub-specialty hepatopancreatobiliary surgeons.

Investigators carried out a survey of all surgeons practicing across the Caribbean and also retrospectively examined records from operating theatres in each of these countries from July 1, 2013 to June 30, 2017. Any patient treated with a completion cholecystectomy for a symptomatic gallbladder remnant was identified. A patient was considered to have a symptomatic gallbladder remnant when they presented with gallstone-related symptoms, had a reliable history of cholecystectomy (confirmed on histopathology or operative notes), and had a gallbladder reservoir, regardless of its size, confirmed to be present on magnetic resonance cholangiopancreatography (MRCP). 

The hospital records for these patients were retrieved and analyzed retrospectively over the four-year study period. The following data were extracted: patient demographics, symptomatology, results of imaging, indications for operation, interval between initial cholecystectomy and re-presentation, intraoperative details, surgeon details, surgical techniques, specialized equipment, conversions, morbidity, and mortality at completion operations. Data were entered in a Microsoft Excel worksheet and descriptive statistics were generated using Statistical Packaging for Social Sciences (SPSS), version 12.0 (SPSS Inc., Chicago IL).

## Results

Over the four-year study period, 12 completion cholecystectomies were performed across the Anglophone Caribbean. The completion operations were performed in Trinidad & Tobago (7), Jamaica (4), and the Cayman Islands (1). No completion operations were reported from any other Caribbean country over the study period. 

All patients had a previous attempt at cholecystectomy - seven by a laparoscopic approach and five attempts were at open surgery. Table 1 outlines the indications for the original operations.

**Table 1:** Patients having completion cholecystectomy in the Caribbean

Table 1: Patients having Completion Cholecystectomy in the Caribbean							
No	Sex	Age	BMI	Approach to original operation	Urgency of operation	Indication for original operation	Partial Cholecystectomy
1	F	57	28	Open	Elective	Biliary Colic	Un-intentional
2	M	52	32	Laparoscopic	Emergent	Gallbladder Empyema	Deliberate
3	F	60	26	Open	Emergent	Acute Cholecystitis	Un-intentional
4	F	52	28	Laparoscopic	Elective	Chronic Cholecystitis	Un-intentional
5	F	48	36	Laparoscopic	Emergent	Gallbladder Empyema	Deliberate
6	F	48	29	Laparoscopic	Elective	Chronic Cholecystitis	Un-intentional
7	F	46	29	Mini-open	Emergent	Acute Cholecystitis	Un-Intentional
8	F	40	32	Laparoscopic	Elective	Biliary Colic	Un-intentional
9	M	50	30	Open	Emergent	Gallbladder Empyema	Deliberate
10	F	45	27	Laparoscopic	Elective	Chronic Cholecystitis	Un-intentional
11	F	32	35	Laparoscopic	Elective	Biliary Colic	Un-intentional
12	F	39	38	Mini-open	Elective	Chronic Cholecystitis	Un-intentional

Interestingly, seven of the original cholecystectomy attempts were elective operations for biliary colic or chronic cholecystitis. A review of these operative notes suggested that the operative procedures in these cases were uneventful and a sub-total cholecystectomy was not deliberately performed. 

Five index cholecystectomies were performed on an emergent basis for gallbladder empyema or acute cholecystitis. In three of these cases a deliberate decision was made to perform a partial cholecystectomy. In one case (#2), during an emergency laparoscopic cholecystectomy for a gallbladder empyema, the surgeon encountered bile spilling from what was thought to be a BDI. This was intubated with a T-tube. However, a post-operative T-tube cholangiogram revealed it was in fact a gallbladder remnant with a large stone. During two emergent cholecystectomies for gallbladder empyema (cases #5 and #9), partial cholecystectomy deliberately performed because the surgeon was concerned about the potential for BDI due to inflammatory changes at Calot’s triangle. 

At the time of re-presentation, the patients had an average age of 48.2 years (standard deviation (SD) 7.7; range 32-60; median 48), a mean body mass index (BMI) of 30.8 Kg/M2 (SD 3.81; range 26-38; median 29.5) and a 6:1 female preponderance. These patients all re-presented with gallstone-related symptoms: acute cholecystitis (7), gallstone pancreatitis (3), and post-cholecystectomy pain syndrome (2). The average interval between the index operations and completion operations was 14.8 months (SD 12.33; range 1-48; median 13).

Completion operations were all done on an elective basis. Three patients who re-presented with severe biliary pancreatitis had their operations performed after an average delay of six weeks. In all cases, a laparoscopic approach was used to perform completion operations - despite the original operations being open in five cases. 

The intra-operative experience was similar in all cases, with dense adhesions in the right upper quadrant that precluded visualization of the remnant. In all cases, the adhesions tethered the stomach, duodenum, and/or transverse colon to the gallbladder remnant. Meticulous adhesiolysis was needed to identify the tip of the remnant and retrograde dissection of structures in Calot’s triangle was not possible in any case. Therefore, in all cases, a dome-down technique was used. 

Intra-operative cholangiography was performed in 7 (58%) cases to assist with the identification of difficult anatomy. In three cases, cautery attached to “hot scissors” was used to perform the operations and ultrasonic dissectors were used in the remaining nine cases. 

There were no conversions in this series and in all cases, dissection down to the cystic duct and artery - although difficult - was possible. In all cases, sufficient dissection could be done to demonstrate Strasberg’s critical view and the cystic duct and artery could be individually ligated with clips. 

These operations were completed with an average blood loss of 135ml (SD 101.5; range 40-400; median 120) and a mean operating time of 126.5 minutes (SD 29.4; range 90-180; median 122.5). A minor complication occurred in one (8.3%) patient using the modified Clavien-Dindo classification [9]. This was a prolonged bile leak in a 48-year-old woman. In this case, an intra-operative drain was left in situ at the gallbladder bed and the leak resolved spontaneously within 10 days of operation without further intervention. No major complications or deaths were reported. 

In this series, the mean hospital stay was 2.4 days (SD 1.3; range 1-5; median 2.5) and patients returned to work on average 10.4 days (SD 6.0; range 5-21; median 8.5) after the operation. 

These patients were followed for a mean duration of 73.25 months (range: 36-112; median 73.5; SD +/- 25.82). During this period of follow-up, there were no complications or re-admissions directly related to the completion cholecystectomies. Additionally, during the surveillance period, there were no patients with continued symptoms attributable to gallbladder remnants and no incisional hernia was detected. 

## Discussion

Most surgeons’ fallback position when they experience difficulty dissecting Calot’s triangle is to perform a partial cholecystectomy [1-5,10]. We agree fully that this is the safe option when the surgeon is concerned about the risk of BDIs. However, we also recognize that there is a potential for these patients to develop pathology in the gallbladder remnant [4-,7,10]. A completion cholecystectomy then becomes necessary to prevent further episodes [4-7]. In our series, 25% of persons with remnants developed severe gallstone pancreatitis that carries a significant mortality risk. Since gallbladder remnants may cause life-threatening consequences, we are strong advocates of completion cholecystectomy. 

The true incidence of symptomatic gallbladder remnants remains unknown. Naraynsingh et al. [11] reported that the incidence of symptomatic gallstones in Caribbean was 2.2 per 1000 population. For a Caribbean population of 6,426,914 persons, assuming that all symptomatic patients are treated surgically, we estimated that there should be 14,139 cholecystectomies performed each year across the Anglophone Caribbean. Considering that twelve cases were identified over the four-year study period, there should be one patient with a symptomatic gallbladder remnant who will require completion cholecystectomy for every 4,713 (0.02%) unselected cholecystectomies performed across the Caribbean. This was consistent with reports in the literature that documented symptomatic remnants after 0.02%-5% of unselected cholecystectomies [12-14]. The incidence is even greater when a partial cholecystectomy is deliberately performed, ranging from 4.19% [13] up to 13% [15]. 

In our series, 83% were women at an average age of 48 years. This was similar to most reports where middle-aged women comprise the majority of patients with post-cholecystectomy syndromes from gallbladder remnants [6,7,12,16]. One would expect the numbers to rise in the era of laparoscopy because surgeons would fear BDI due to the lack of tactile feedback and two-dimensional vision at laparoscopy. However, we were surprised to learn that 42% of the remnants that we had to address were left at open surgery. Interestingly, some reports in the literature also bore out this relationship where open surgery was responsible for symptomatic remnants in 52.4% to 100% of cases [12,14]. It is tempting to think that this might be due to operator inexperience, as has been demonstrated repeatedly in the literature [3,12,14,17]. El Nakeeb et al. also noted that 10% of remnants occurred in patients with anatomic anomalies [12]. Therefore, it is important to be aware that there is a greater than expected incidence of hepato-biliary anomalies in patients of Caribbean extract [18-21]. 

Eleven (91%) patients in our series confidently reported that their symptoms were similar to those for which they first had cholecystectomy. This was independent of the interval between index and completion cholecystectomies that varied widely in our series, from as early as four weeks to as late as 12 years. Again, this mirrored reports in the literature, where the interval between index and completion cholecystectomy ranged from two months [12] to 40 years [22]. Therefore, a high index of suspicion should be maintained in patients with post-cholecystectomy pain syndromes and they should be thoroughly investigated for a gallbladder remnant. 

We relied on MRCP to definitively identify gallbladder remnants, with 100% accuracy in our series. This is similar to the literature where MRCP is reported to identify gallbladder remnants with 92% [13] to 100% [12] overall accuracy. That is better than trans-abdominal ultrasound that is reported to identify remnants with 60% [13] to 71.4% [12] overall accuracy. Endoscopic ultrasound is useful to identify small gallbladder remnants [6,12,14,16], but it is not readily available in developing countries. We considered MRCP mandatory to identify remnants, exclude choledocholithiasis, and evaluate biliary anatomy pre-operatively.

Patients with symptomatic remnants require completion cholecystectomy as definitive treatment [4-7] but there has been debate in the literature whether these complex operations should be attempted via the open or laparoscopic approach. In fact, as recently as 2013, when we prepared our first case report of a laparoscopic completion cholecystectomy [5], the manuscript was rejected because a reviewer thought it was unethical to attempt a laparoscopic completion cholecystectomy since the morbidity profile was unacceptably high. This reviewer’s response demonstrated that there was still deep-rooted skepticism toward the laparoscopic approach.

However, a closer look at the literature revealed the first recorded laparoscopic completion cholecystectomy was in 1995 by Gurel et al. [23] in a patient with a remnant causing Mirizzi’s syndrome. Since then, many other small case series have shown that the laparoscopic approach is feasible and accompanied by similar benefits to elective cholecystectomy [4-6,14,16,17,24-27]. A collective analysis of the three largest published series by Parmar et al. [14] (40 cases), Chowbey et al. [17] (26 cases) and El Nakeeb et al. [12] (21 cases) revealed that conversions were required in 1.25% of 80 completion cholecystectomies attempted laparoscopically. In our series, all operations were completed laparoscopically, adding to the literature in support of the feasibility of the minimally invasive approach to completion cholecystectomies.

In all cases, we encountered difficult anatomy that made the operations technically challenging. Many other authors also reported difficult procedures due to unclear anatomy [12-14,23-27] and dense adhesions tethering the colon [12,27], duodenum [12,14] and/or stomach [25-27]. This explains the propensity for these organs to be injured during the dissections [12,14,27]. To achieve safe dissection, we advocate meticulous dissection techniques and judicious use of energy devices. In addition, we used other previously described techniques to reduce the potential for BDI [1]; we advocate their use in these cases: identification of Rouviere’s Sulcus, dome-down dissection, judicious use of energy devices, attaining Strasberg’s critical view prior to transection, liberal use of cholangiography, intra-corporeal suturing of the cystic duct junction, and hepatic drainage.

Our mean operating time (126 minutes) was comparable to existing reports that ranged from 62 minutes [17] to 127 minutes [12]. In our series, there were no major BDIs, but minor bile leaks occurred in 8.3% of cases. This compared well to published results, where major BDI occurred after 2.5% [14] to 4.8% [12] of completion cholecystectomies and minor bile leaks occurred in 7.7% of cases [17]. The morbidity profile of completion cholecystectomies in our hands was good, but it is necessary to disclose that these operations were all performed by subspecialty-trained surgeons. We believe that these complex operations should be reserved for appropriately trained surgeons with advanced laparoscopic experience. This has been shown to be a statistically significant predictor of good outcomes when the laparoscopic approach is used for other difficult cases, such as complicated acute cholecystitis [1,28].

Kohn et al. [29] even advocated robotic completions cholecystectomies because the advantages of the surgical robot could increase the safety profile of these difficult operations. Kohn et al. [29] reported a small series of three robotic completion cholecystectomies with no conversion, morbidity, or mortality. Although their position is easy to understand when one considers the advantages of a surgical robot, there is currently insufficient available data at this point to form an evidence-based recommendation on robotic versus laparoscopic completion cholecystectomy. 

## Conclusions

Laparoscopic completion cholecystectomy can be completed safely with good outcomes and acceptable complications rates. The patients derive the same advantages as elective cholecystectomies. Therefore, laparoscopic completion cholecystectomy performed by hepatobiliary surgeons with advanced laparoscopic expertise in specialized centers should be the new standard of care.
